# It Is All One-Sided: Monocular Blindness and Charles Bonnet Syndrome

**DOI:** 10.7759/cureus.92148

**Published:** 2025-09-12

**Authors:** Wesam Albqaeen, Alexandra C Davies, Gek Shim

**Affiliations:** 1 Stroke Medicine, University Hospital of North Durham, Durham, GBR; 2 Medicine, University Hospital of North Durham, Durham, GBR

**Keywords:** charles-bonnet syndrome, crao (central retinal artery occlusion), unilateral vision loss, visual disturbance, visual hallucinations

## Abstract

We present the case of a 72-year-old male who developed Charles Bonnet Syndrome (CBS) following a right retinal artery occlusion.

Complex visual hallucinations are the main symptom of CBS in persons with substantial vision loss. It has been conventionally associated with bilateral severe impairment, but recent observations indicate under-recognised monocular cases that offer equally notable incidences of CBS.

This case highlights the importance of recognising that CBS can also occur in monocular blindness and its underdiagnosed prevalence in current literature. In order to avoid the misidentification of CBS in unilateral vision loss and to empower patients with information and empathy, increased focus on recognising monocular CBS and providing targeted guidance for patients is required.

## Introduction

Charles Bonnet syndrome was first described in 1760 by Charles Bonnet and is defined as complex visual hallucinations occurring secondary to visual loss with retained insight into the fictionality of the hallucinations [1]. Routinely, individuals experience formed, complex, persistent or repetitive, stereotyped visual hallucinations, typically with retained insight [2]. CBS is a common condition, estimated to have a prevalence of 10% among ophthalmology patients, but it is thought to be grossly underreported due to limited awareness of the condition by physicians and patients [2].

Visual hallucinations can be distressing to patients, particularly when patients are not aware that hallucinations can be caused by their visual loss; visions are particularly disconcerting, as individuals may fear they are suffering from a severe mental health condition. This can cause patients to conceal symptoms from physicians and medical professionals due to concerns they will be labelled as mentally unwell and fears associated with related stigmatisation [2]. Clinicians are also often unaware of CBS and its relevance to their patients. As a consequence, patients are often not informed and educated about the fact that they may suffer from visual hallucinations as a result of their visual impairment.

Charles Bonnet syndrome may present in any condition with an impaired visual pathway, but is most commonly defined as loss of vision exceeding 60% [3]. According to Forte et al., CBS is most frequently a product of bilateral severe vision impairment [4]. This definition is reflected in current literature, where studies and case reports predominantly focus on bilateral visual defects, and monocular visual pathologies are often overlooked. There are increasingly more frequent case reports and literature reviews describing the prevalence of CBS after monocular vision loss [5], challenging this definition and broadening the diagnostic scope for patients with a wider range of visual loss. This case presentation discusses this less recognised phenomenon, to add to the increasing awareness among clinicians and thus improve outcomes for patients.

## Case presentation

A 72-year-old male presented to the emergency department with sudden-onset visual changes in his right eye. Visual disturbances were described as flashing lights like fireworks. Following this, 20 minutes later, he experienced a black curtain descending from the top of his vision down, extending across the full vision. He reported a lack of vision in his right eye, which persisted without improvement. 

He was initially referred by the emergency department to ophthalmology, who reviewed him on the same day in the urgent care clinic. Visual assessment demonstrated light perception only in the right eye and acuity of 6/24 unaided in the left eye, improving to 6/5-3 with correction. Fundoscopy demonstrated central retinal artery occlusion with a cherry red spot at the macula in the right eye, and no abnormalities were detected in the left eye. There was no temporal artery tenderness or thickening of the temporal arteries bilaterally, no diabetic changes or cataracts in either eye. He was diagnosed with central retinal artery occlusion (CRAO) and was discussed with the on-call stroke consultant, given 300 mg of aspirin and referred to the stroke team for assessment within 24 hours. He was assessed by the stroke team and commenced on stroke secondary prevention. On assessment with the stroke team, a right-sided hemianopia was detected, and he was referred to orthoptics for further visual assessment.

**Figure 1:**
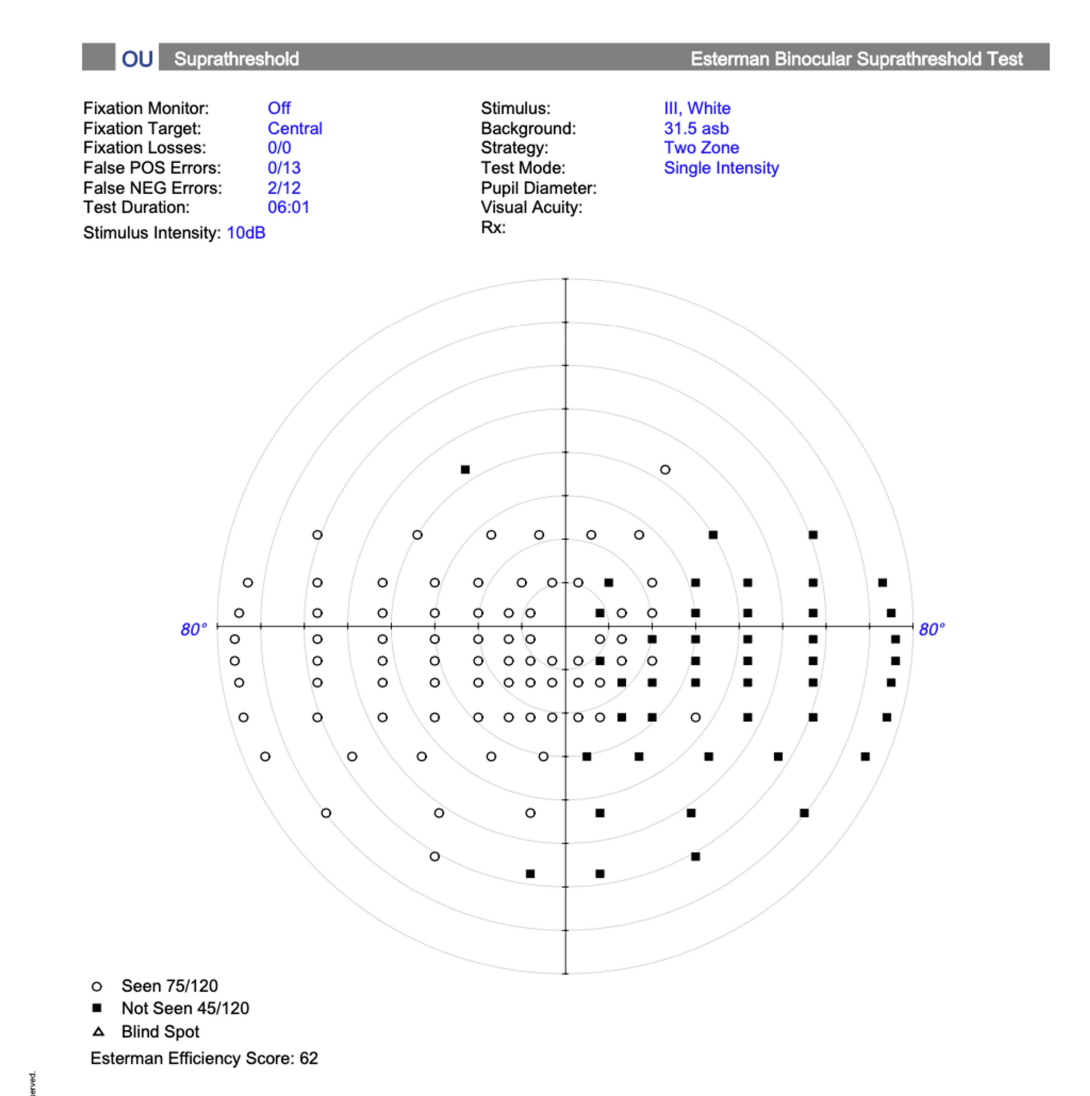
Visual field assessment

Visual assessment by orthoptics demonstrated no perception of light in the right eye and a relative afferent pupillary defect. Initial binocular Esterman visual field testing (Figure 1) appeared to show a right homonymous hemianopia. This pattern was explained by two factors: (1) profound vision loss in the right eye from central retinal artery occlusion (CRAO), and (2) obstruction of the nasal field of the unaffected left eye by the patient’s prominent nasal bridge during binocular testing, mimicking a midline-respecting defect. Monocular testing confirmed that the deficit was confined to the right eye. CT brain showed no acute intracranial abnormality, supporting the absence of an additional central visual pathway lesion.

The patient had no previous history of visual symptoms, headaches or mental health disorders. His past medical history included conditions increasing his risk of cardiovascular events, including previous myocardial infarction, ischaemic heart disease, polycythaemia rubra vera, hypertension, diabetes mellitus type 2, chronic kidney disease and non-alcoholic liver disease.

On follow-up with the stroke team, six weeks after his initial presentation, the patient described ongoing right monocular visual loss; he also volunteered that he was experiencing visual hallucinations, which appeared to be typical of CBS. He described visual hallucinations with no auditory or sensory elements occurring daily, more frequently in dim light, and the hallucinations did not interact with him. He retained insight into the fictionality of these hallucinations but was distressed by their occurrence. The hallucinations were initially very intense in nature, but this intensity reduced after the first month. The hallucinations occurred when his eyes were open and disappeared when his eyes were closed. He described a few key recurring hallucinations typical of CBS, including small floating shapes and animals, such as a lizard moving across his vision, often appearing on blank surfaces or in dim light.

As stated above, the patient was always aware that the hallucinations were not true images, but was not aware that hallucinations could happen with his visual loss, and so was distressed by them. He did not understand what they were or why they were happening. Increased awareness of the diverse manifestations of CBS, including in monocular visual loss, would have enabled earlier recognition in this patient. After educating the patient about CBS, its manifestations and disease course, his distress was somewhat relieved through understanding his symptoms and their cause.

## Discussion

Selected cases describing unilateral CBS, including their causes, visual experiences, and clinical significance, are presented below in the literature review. These cases demonstrate that even partial vision loss warrants awareness and careful diagnosis to reassure and reduce distress in patients.

Charles Bonnet syndrome has been observed as a widespread condition among patients with severe visual impairments. A comprehensive systematic review found the prevalence of CBS in patients with ophthalmic diseases estimated to be 10%, with the condition being usually underestimated in clinical practice [2]. According to Rojas and Gurnani, key reasons for this are patients failing to report their symptoms due to fear of being stigmatised and misjudged [6]. These observations suggest that CBS is not a rare condition isolated to a minority of people, but a common and primary characteristic of vision loss that clinicians should be aware of. Environments where structured questioning and patient education are applied, including low-vision rehabilitation clinics, presented higher detection rates, highlighting the importance of empathetic medical inquiry in effective identification of CBS [2].

Recent case reports have contested the long-held notion that bilateral profound loss of sight is a precondition for the development of CBS. It has been observed that vivid hallucinations may occur in patients undergoing enucleation as a treatment for intraocular tumours. For instance, enucleation for choroidal melanoma has led to complex visual scenes confined to the blind field, illustrating that unilateral sensory deprivation can trigger cortical hyperexcitability [7]. Optic neuritis can produce similarly intense symptoms, suggesting that inflammatory optic nerve damage disrupts inhibitory processes [5]. Central retinal artery occlusion and rubeotic glaucoma have also been linked to persistent hallucinations of geometric shapes or moving figures, highlighting how both acute and chronic unilateral deficits can induce CBS [5].

Optic neuritis and retinal artery occlusion are other instances characterised by extravagant visual images and complicated visualised events, though preservation of partial visual capabilities in the other eye occurs [8]. The awareness of unilateral CBS management and specifics, as well as careful utilisation of systematic assessment, enable clinicians to treat CBS-related hallucinations with confidence and empathy.

The mechanism underlying Charles Bonnet syndrome is thought to be caused by the shifts in the visual cortex after sensory deprivation. According to the deafferentation hyperexcitability model, partial damage to visual input affects inhibitory messages and leads to spontaneously fired neural impulses, which the patients take as multilevel images [7]. Cleveland Clinic has suggested that in unilateral cases, the non-affected eye serves adequately as a visual stimulus to override hallucinations [9], but clinical experience, such as this case study, shows that such compensation is not always complete and may prompt further difficulties. This implies that cortical hyperactivity can be precipitated by deafferentation even of a limited degree and that there is a continuum in vision loss rather than a definitive threshold [10]. The knowledge of this process plays an essential role in explaining the occurrence of CBS even in cases of partial maintenance of visual abilities.

Successful diagnosis of CBS are achieved by being knowledgeable of specific diagnostic criteria and understanding diverse manifestations that may easily be confused with other conditions. The prominent symptoms are intricate, developed visual hallucinations prompted by visual impairment when the cognitive and insight processes are preserved [7].

As visual hallucinations are also associated with unrecognised psychiatric or neurodegenerative disorders, one should deeply comprehend the differences and diagnostic measures so as not to confuse the patients and avoid frequent misdiagnosis [7]. Reassurance and education of patients are the first-line interventions in the management of confirmed CBS, which effectively alleviates anxiety and enhances coping [2].

## Conclusions

Overall, the presentation and discussion of this case demonstrate that CBS can occur in monocular visual loss, challenging the long-held notion that bilateral profound loss of sight is a precondition for the development of CBS. It, therefore, adds to the literature evidencing this phenomenon, increases awareness of CBS in monocular vision loss and broadens its diagnostic scope. Given that patient education and reassurance are treatments for CBS, they have been shown to effectively enhance coping. Increased awareness among physicians and medical professionals of the diverse manifestations of CBS, including in monocular visual loss, as in this case study, provides an opportunity for improved care in CBS.

## References

[1] Bonnet C (1782). Essai Analytique Sur Les Facultés Dâme [In French].

[2] Christoph SE, Boden KT, Siegel R, Seitz B, Szurman P, Schulz A (2025). The prevalence of Charles-Bonnet syndrome in ophthalmic patients: a systematic review and meta-analysis. Brain Res Bull.

[3] Potts J (2019). Charles Bonnet syndrome: so much more than just a side effect of sight loss. Ther Adv Ophthalmol.

[4] Forte G, Assaf N, Forte P, Jolly JK (2025). Charles Bonnet syndrome associated with unilateral vision loss: a new diagnostic perspective. Ophthalmic Physiol Opt.

[5] Tripathy K SS, Waymack JR (2025). Central retinal artery occlusion. StatPearls [Internet].

[6] Rojas LC, Gurnani B (2025). Charles Bonnet syndrome. StatPearls [Internet].

[7] Woods MD, Siliezar PD, Laylani N, Leitão M, Lee AG (2024). Monocular Charles Bonnet syndrome secondary to optic neuritis and the utilization of artificial intelligence to illustrate visual hallucinations. Can J Ophthalmol.

[8] (2025). NHS. Charles Bonnet syndrome. https://www.nhs.uk/conditions/charles-bonnet-syndrome/.

[9] (2025). Cleveland Clinic. Hallucinations. https://my.clevelandclinic.org/health/symptoms/23350-hallucinations.

[10] daSilva Morgan K, Collerton D, Firbank MJ, Schumacher J, ffytche DH, Taylor JP (2025). Visual cortical activity in Charles Bonnet syndrome: testing the deafferentation hypothesis. J Neurol.

